# First referral hospitals in low- and middle-income countries: the need for a renewed focus

**DOI:** 10.1093/heapol/czad120

**Published:** 2023-12-20

**Authors:** Rosanna Jeffries Mazhar, Tamara Mulenga Willows, Suraj Bhattarai, Chit-Su Tinn, Nadine Misago, Mike English

**Affiliations:** Health Systems Collaborative, Centre for Global Health Research, Nuffield Department of Medicine, University of Oxford, Peter Medewar Building, South Parks Road, Oxford OX13SY, United Kingdom; Health Systems Collaborative, Centre for Global Health Research, Nuffield Department of Medicine, University of Oxford, Peter Medewar Building, South Parks Road, Oxford OX13SY, United Kingdom; Department of Clinical Research, London School of Hygiene & Tropical Medicine, Keppel Street, London, WC1E 7HT United Kingdom; Department of Global Health, Global Health Research & Medical Interventions for Development (GLOHMED), 99 Shree Marg, Lalitpur, Kathmandu 44700, Nepal; Faculty of Social Sciences and Humanities, Mahidol University, 999, Phutthamonthon Sai 4 Road, Salaya, Nakhon Pathom 73170, Thailand; Centre for Tropical Medicine and Global Health, Nuffield Department of Medicine, University of Oxford, Peter Medewar Building, South Parks Road, Oxford OX1 3SY, United Kingdom; Interdisciplinary Research Group in Public Health, Doctoral School, University of Burundi, Avenue de l’Unesco N° 2., Bujumbura 1550, Burundi; Health Systems Collaborative, Centre for Global Health Research, Nuffield Department of Medicine, University of Oxford, Peter Medewar Building, South Parks Road, Oxford OX13SY, United Kingdom; Health Services Unit, KEMRI-Wellcome Trust Research Programme, 197 Lenana Rd, Nairobi P.O Box 43640-00100, Kenya

**Keywords:** First-level hospitals, referral hospitals, first referral hospitals, district hospitals, hospital health systems

## Abstract

First referral hospitals (FRHs) are the hospitals closest to the community, which offer expertise or technologies to complement more widely available ‘basic’ ambulatory care or inpatient care. Despite having been a subject of interest in global health policy in the latter half of the 20th century, in more recent decades, they appear to have been overshadowed. This paper reviews what is understood by FRH, drawing on both academic and policy literature, complemented by specific country case studies. We undertook three reviews: a grey literature review of global and regional policy reports and documents, a structured review of the academic literature on FRH and a review of FRH-related policies in eight countries. Our findings indicate that there is confusion regarding the definitions and roles of FRH; they have fallen off the policy agenda globally and they suffer from lack of advocates in part related to the absence of cohesive definition. Meanwhile, these facilities continue to fulfil important functions in health systems in low- and middle-income countries, and expectations for service delivery remain high. In light of these findings, this paper calls for renewed interest and investment in FRH from the global health academic and policy-making community.

Key messages

- First referral hospitals (FRHs) are poorly defined at global and regional levels and require clearer thinking to inform health system policies, planning and resource allocation.
- Despite their importance, which was recognized in the latter half of the 20th century, FRHs now lack advocates and could be considered neglected having fallen off the global health research and policy agendas in the recent decades.
- Irrespective of the global context, at the country levels, FRHs are expected to, at varying levels, provide essential health services and fulfil important roles in public-sector health systems.

## Introduction

Hospitals’ roles within health systems, both as imagined and as implemented, have evolved over time. By the end of the 19th century, advances in anaesthetics, surgery and other medical specialities encouraged hospitals to provide increasingly sophisticated care. In this process, hospitals became relatively isolated from other health institutions and activities, contributing to a predominantly disease-focused model of health care at the time (WHO, 1987b). In the 1920s, first attempts at dividing the healthcare system occurred, introducing the term ‘primary care’, which broadly aligned with clinic-based health care, and ‘secondary’ and ‘tertiary care’, which encompassed increasing degrees of specialized hospital care (Frenk, 2009). This model, however, was reviewed and challenged throughout the second half of the 20th century, during which the roles of hospitals became a matter of considerable focus in global health policy spheres, and criticisms of the hospital-centric approach emerged.

In 1956, World Health Organization (WHO) reviewed the organization of medical care and agreed that hospitals cannot work in isolation, proposing that hospitals should play a role in preventive activities, in addition to their primary curative functions. Hospitals’ roles in providing the so-called ‘extra mural care’ was re-emphasized in a report by the same committee in 1959 (WHO, 1987b). The Alma Ata Declaration of 1978, which promoted primary health care (PHC) as the key to attaining ‘Health for All’, represents a momentous and historical shift in focus away from previous hospital-centred care models. While hospitals were not the focus of Alma Ata, it reiterated hospitals’ public health supportive roles in line with the discourse at the time (WHO, 1987b; Chabrol *et al.*, 2018).

Over time, different terms began to emerge to differentiate and describe first referral hospitals (FRHs). In 1981, a WHO conference to review hospitals’ roles in PHC used the term ‘hospitals at the first referral level’ (WHO, 1992a), describing these facilities’ role as the first referral point from a preceding level within the health system. In 1986, WHO incorporated the role of hospitals at the first referral level into a proposed district health system model and begot the term ‘district hospital’. These were envisaged as the only hospitals at the first referral level within a district health system, which they defined as health services organized by and managed for the benefit of overseeing health in an administrative district, in line with the common administrative hierarchies at that time (WHO, 1992a). Nearly 20 years later, in the third edition of the Disease Control Priorities Project (DCP3), the term ‘first level hospital’ is used to indicate the first hospital within the health system providing access to essential surgery (McCord *et al.*, 2015). Throughout this paper, we adopt the term ‘first referral hospital’ to denote the first hospital/s in the hierarchy of government health systems that offer higher levels of diagnostic and management expertise for more complex acute or chronic conditions. This is primarily for the sake of simplicity, and our intention is not to discredit various terms used in different settings.

The policies and meetings that introduced first referral level and district hospital terminology also attempted to define the roles and function of FRH. In 1985, WHO stated that hospitals at the first referral level should provide a ‘fully comprehensive range of promotive, preventive, curative, and rehabilitative health activities’ to reach the communities which they serve. The roles of these hospitals within a district health system were re-emphasized, towards supporting all elements of PHC programmes in an integrated manner (WHO, 1987b). In 1990, WHO clarified that district hospitals should ‘support’, not deliver, PHC (WHO, 1990), and in 1992, they defined for the first time the expected diagnostic, treatment, counselling and rehabilitation services of hospitals at the first referral level (spanning family medicine and PHC, [internal] medicine, obstetrics, mental health, eye care, rehabilitation, surgery, paediatrics and geriatrics) (WHO, 1992b).

Thus, by the end of the 20th century, the perceived roles of FRH as distinct service delivery platforms had transformed. Instead of being curative-focused health facilities, serving just the advanced health needs of some of the very ill, a larger scope of functions was envisaged for FRH. Their role would encompass promotive, preventive, curative and rehabilitative health services, central to supporting the PHC aims and the district health model polices. The tone of policy documents from this time was optimistic, despite numerous stated challenges.

However, attaining sustained health system change is a complex task. Several decades have passed since the FRH policies described earlier were promoted, and it is unclear whether this global vision of the expanded role of FRH in support of PHC has been embraced and applied across low- and middle-income countries (LMICs) today. Our experience was that confusion around the meaning of PHC, and hospitals including FRH placement or roles therein, persists. These are important issues. The need for expertise and technologies that are often restricted to hospitals is growing globally, in line with growing chronic disease burdens. The recent COVID-19 pandemic revealed performance issues across the hospital sector. Both in high- and low-income settings (Chabrol *et al.*, 2018; WHO, 2022c), much needed light was shone on the importance of functional and effective management and referral of the critically ill.

In this context, this paper examines the authoritative policy documents and academic literature to determine how FRHs are currently understood and operationalized at the global, regional and national level, with an aim to uncover whether there is a cohesive understanding of FRH. This is the first review of this subject that aims to comprehensively establish the current status quo of FRH.

## Methods

Given the broad nature of our research question, a systematic review was deemed neither appropriate nor feasible. Instead, we undertook a narrative review, with an aim to provide a comprehensive narrative synthesis of the overall body of literature on the subject of FRH. We included the following three distinct sources of information: (1) academic literature on FRH, (2) grey literature including global and regional policy reports and documents and (3) FRH-related policies in eight-country case studies. Our approach to each is described below. Regarding the scope of our review, although we recognize that private sector hospitals do play important roles within countries’ health systems, our focus was on public sector facilities which tend to be better described and fall under the remit of governments’ authority and responsibility.

### Structured academic literature review

To understand contributions from the research community on FRH, we undertook a two-part review of the academic literature (summarized in detail in supplementary material 1). Part 1 aimed to determine what recommendations are made on FRH-expected clinical services in LMICs by screening published academic papers for content on FRH definitions and service expectations. To this end, we searched the following databases on 3 August 2022 with no limits for language or publication dates, based on specific search terms (see supplementary material 2): Ovid Embase; Ovid Medline; Ovid Global Health; Ovid PsycINFO; Ovid AMED; EBSCOhost CINAHL; the Cochrane Database of Systematic Reviews; the Cochrane Central Register of Controlled Trials. To ensure completeness in our review, we supplemented this structured search by purposively including all articles published in *The Lancet* Commissions and Series and as well as several additional papers with potential relevance to FRH that had been kept in a pre-existing repository by one of the authors. In total, we screened 2703 papers, full-text reviewed 126 and extracted data on FRH definitions and expected services from 16 articles, using a standardized excel-based data extraction tool.

Part 2 of the academic literature review aimed to further understand and characterize the academic research landscape on FRH. To this end, we sought to identify papers for which FRH was the ‘main focus’ of research, by searching for articles specifically mentioning FRH or district hospital in their titles only (see search terms in supplementary material 3). We screened title and abstract of all 132 identified articles and categorized them thematically using excel, based on iteratively developed ‘research-type’ categories.

### Grey literature review

Two reviewers conducted a purposeful review of the policy literature on FRH. Given their role as the normative global agency on health, we focused on WHO reports and documents including those from headquarters and the six WHO regional offices. WHO websites, libraries and institutional repository for information sharing (IRIS), which houses all policy documents that have been published for the last few decades, were our primary source for documents included. Our key search terms were ‘first level hospitals’, ‘first referral level hospitals’, ‘hospitals at the first referral level’, ‘district hospitals’ and ‘hospitals’. We searched United Nations Children Fund (UNICEF) and United Nations Population Fund (UNFPA) libraries with the same search terms, but did not find any relevant documents, likely due to non-specificity of search functions. We also reviewed the World Bank DCP editions, as well as the World Health Assembly decisions and resolutions over the past 15 years. Documents were identified through snowballing method, until reviewers reached saturation point where no new relevant documents were being unearthed (determined through consensus). We summarized papers and extracted relevant text into word documents, which we further synthesized for thematic analysis.

### Case studies

Our eight-country case studies include Myanmar, Vietnam, South Africa, Sri Lanka, Rwanda, Burundi, Papua New Guinea and Nepal. We purposively selected these based on our team’s location and ability to access and read the information sources in the languages they are published in, with an attempt to encompass different levels of development and regions of the world. Countries were assigned to individual reviewers who searched government websites and used internet search engines for documents that described hospital care and health system policies (see supplementary material 4 for list of documents reviewed). Myanmar, Vietnam, Burundi, Rwanda and Nepal case studies were completed with the help of in-country health professionals. Further documents were identified through snowballing method, until reviewers reached saturation point where no new relevant documents were being identified. Relevant information was extracted for each country, using a standardized Microsoft word template (see supplementary material 5) from which we created a summary table in excel for comparative analysis.

## Results

We triangulated findings from our three information source to iteratively derive the following thematic observations: (1) there is no cohesive understanding of FRH within existing policy, (2) FRH have fallen off the policy agenda, (3) there is scarce academic research or advocacy for FRH and, despite this, (4) expectations of FRH as service delivery platforms remain high. We now deal with each of these issues in turn, drawing on results from our various sources as relevant to each theme.

### There is no cohesive understanding of FRH within existing policy

There is considerable variation regarding FRH naming and classification within health systems, evident across all information sources we reviewed. This suggests confusion.

In the academic literature, we identified different terms used to denote FRH, including district hospital, first-level hospital, secondary care facilities, local hospital, level 1 hospital and first-level referral hospitals. In the few papers that attempted to define FRH, we noted ambiguity regarding categorization vis-à-vis primary care (Table 1). While some equate FRH as ‘secondary level’ care providers (Bukhman *et al.*, 2015; Boudreaux *et al.*, 2022), with one paper stating they are the lowest level of care in secondary facilities (Boudreaux *et al.*, 2022), another describes them as gatekeepers to higher level (secondary or tertiary) care (Ng-Kamstra *et al.*, 2016), suggesting they fall outside of these, presumably within primary care. Academic papers differed with regard to target population sizes to be served by district hospitals with a range of 100 000–500 000 in one, 250 000 in another and 100 000–one million in another.

**Table 1.** Definitions of FRH from academic literature

Definition	Source
First referral-level hospital or the district hospital provides a level of care that cannot be obtained at home; acts as a gatekeeper for referral to higher levels of care at a secondary or tertiary hospital (Ng-Kamstra *et al.*, 2016).	Global Surgery 2030: evidence and solutions for achieving health, welfare and economic development
Secondary facilities, first-level hospitals (otherwise known as DHs, first referral-level hospitals or secondary care facilities) serve populations of around 250 000 people and are often the hubs of district health systems. In many countries, they are considered as part of the primary health-care system (Bukhman *et al.*, 2015).	Reframing non-communicable diseases and injuries for poorest billion
Local hospital, which must provide complementary referral services (notably emergency obstetric and surgical care) and should make its superior resources available to support integrated district wide care (Segall, 2003).	District health systems in a neoliberal world: a review of five key policy areas
In sub-Saharan Africa (SSA), DHs or Level 1 hospitals according to the WHO are the first level of hospital that provide in-patient surgery and anaesthesia. In general, they cater for essential and emergency surgical care for populations of 100 000–500 000 (Bentounsi *et al.*, 2021).	Which surgical operations should be performed in DHs in East, Central and Southern Africa? Results of a survey of regional clinicians
Secondary care facilities are defined as DHs or first-level referral hospitals and noted as the lowest level of care in secondary care facilities (Boudreaux *et al.*, 2022).	Addressing severe chronic NCDs across Africa: measuring demand for the Package of Essential Non-communicable Disease Interventions-Plus (PEN-Plus)
DHs are often located in a district’s capital and can be a central location for medical referrals; training of health workers, including clinical assistants and nurses; supervision of peripheral facilities and public health surveillance. Such hospitals are generally 50- to 200-bed institutions that provide care for a district’s 100 000 to 1 million people (Rajbhandari *et al.*, 2020).	The Neglected Hospital—The DH’s Central Role in Global Health Care Delivery.

Similar discrepancies were noted in the policy literature. While the 2006 DCP2 uses the term ‘district hospitals’, we noted an absence of this term in more recent policy documents, perhaps due to the phasing out of administrative districts in some settings. The third and most recent DCP3 edition refers to first-level hospitals, which they define on the basis of being able to provide specific services (surgery, inpatient care, outpatient specialist care and routine pathology services that cannot be feasibly delivered at lower levels as defined in their essential universal health coverage package), distinct from referral and speciality hospitals (Watkins *et al.*, 2017). We also noted a tendency in more recent policy documents to use the term ‘hospital sector’ or ‘hospitals’ without specific mention of sub-sectors therein. The placement of FRH within health systems (primary vs secondary care) further confuses and is confused by their perceived role within PHC. While earlier editions of DCP described district hospitals as ‘the apex of the pyramid of primary health care’ (English *et al.*, 2006), more recent documents on PHC present hospitals (as a grouped entity) as receiving a skewed proportion of resources (WHO and UNICEF, 2018; Barış *et al.*, 2021) and to some extent competing with PHC (Hanson *et al.*, 2022). This seems to imply that no hospital of any kind is considered part of primary care, and financing for FRH is therefore not seen as contributing to PHC, despite their acknowledged important role in ‘supporting’ PHC (WHO and UNICEF, 2018; 2022).

Among the eight-country case studies, by contrast, the term ‘district hospital’ remains common and is used to denote FRH; however, others include township hospital in Myanmar, Base Hospital in Sri Lanka and Primary/Basic Hospital in Nepal (Table 2). The use of the term ‘district hospital’ does not appear to relate to whether FRHs are embedded within district health systems, as is the case in Vietnam, Papua New Guinea, Burundi and Rwanda. We also observed differences in how FRHs are classified within the health systems with an almost equal distribution between countries referring to them as part of primary and secondary care, and some situating them within both (depending on size and location), which suggests differences exist even within a country. FRH placement within health system hierarchies also differs by country. While in most cases those fulfilling the role of FRH are the hospitals closest to the population that they serve, both Myanmar and Sri Lanka have an additional hospital level below the facility that appears to function as a FRH (station hospitals and divisional hospitals, respectively), and Burundi is introducing this lower tier in the form of communal hospitals. Thus, in some settings there appears to be a distinction between first-level hospitals and FRHs.

**Table 2.** Comparison of country case studies

	Myanmar	Viet Nam	South Africa	Rwanda	Sri-Lanka	Burundi	Papua New Guinea	Nepal
Population	51 100 000	98 168 829	60 041 996	13 000 000	21 909, 000	12 255 429	9 119 005	29 674 920
Economic category	LMIC	LMIC	UMIC	LMIC	LMIC	LIC	LMIC	LMIC
FRH position in health system	Primary and Secondary care	Primary care	Primary and Secondary care	Secondary care	Secondary care	Primary care	Secondary care	Primary care
Term for FRH	Township Hospital	DH	DH (small, medium and large)	DH	Base Hospital (types A and B)	DH	DHs	Primary/Basic Hospital (DH term being phased out)
Any hospitals below FRH?	Yes, station hospitals	No	No	No	Yes, divisional hospitals	Communal Hospital (not yet operational)	No	No
Bed capacity range	25–100	50–200	50–600	Approx. 300	Type A average 307 Type B average 158	75 minimum	50	5–15
Part of the district health system	No	Yes	Yes	No	No	Yes	No	Yes
Defined service package /norms for FRH?	Yes, in draft	No	Yes	Yes	Yes	Yes	Yes	Yes
Defined staffing norms for FRH	No	No	Yes	Yes	No	Yes	Yes	No
FRH services financed by UHC	Yes	Information not found	No	Yes	Yes	Yes for pregnant women and children under 5 years	No	No
FRH mentioned in current National Strategic Health Plan	Yes- service expansion and role in reaching wider population outlined	Information not found	No	Yes	Yes- intention to increase proportion of FRH stated	Yes, set a target to accredit two DHs/year; to integrate rehabilitation services into all DHs; and to construct four new DHs by 2027	Yes- prioritizing medical doctors for DHs included alongside other plans for strengthening the health system	Yes- specific interventions targeted at the DH outlined

### FRHs have largely fallen off the policy agenda

Our review of the policy literature further reveals that, with the obvious exception of DCP3, the focus and perhaps interest in FRH has waned over recent decades at global and regional levels.

WHO’s 2008 report on PHC, aimed at renewing the aims set out in Alma Ata Declaration, reiterates the ‘classic image of the healthcare system based on PHC [as] that of a pyramid with the district hospital at the top and a set of (public) health centres that refer to the higherauthority’. While positioning primary care as the gatekeeper to different forms of referral care, this does not elaborate on the role of FRH within PHC (WHO, 2008). Meanwhile, the recent report by WHO/UNICEF on reimagining PHC in the 21st century (WHO and UNICEF, 2018) makes no reference to the role of FRH. WHO’s current informational webpage on hospitals similarly makes no mention of FRH, but instead alludes in general terms to the importance of hospitals to the attainment of universal health coverage (UHC) in its summary page. The WHO’s ‘District hospitals-guideline for development’ is the only listed document related to FRH specifically; however, this is labelled as ‘old’, with no updated version available (WHO, 2022b). WHO’s global health observatory does include an indicator on the number of district/first-level referral hospitals; however, it lacks any current data, which suggests these data are no longer being tracked (WHO, 2022a).

We reviewed the decisions and resolutions of the World Health Assemblies over the past 15 years (2007–21), which revealed a similar finding. Hospitals were mentioned in several resolutions over this time frame, albeit in relation to specific topics, including trauma/emergency care (*n* = 3), baby-friendly hospital initiative (*n* = 3), prevention and management of pneumonia (*n* = 1), antimicrobial resistance surveillance (*n* = 1) and emergency preparedness (*n* = 1). FRHs, on the other hand, were only mentioned in resolutions in 2014 and 2015, in relation to improving the quality of maternal and new-born care and strengthening emergency and essential surgical care and anaesthesia, respectively (summarized in supplementary material 6).

Beyond those that explicitly mention FRH in their title, we did not systematically review normative guidelines for all medical specialities for content on FRH. However, among the documents that we encountered in our grey literature search, we observed that surgery and maternal and new-born care are among the medical disciplines which most often adopted the terminology of FRH or hospitals at the first referral level. WHO published guidelines on surgical care at the district level in 2003 (WHO, 2003; Lawn *et al.*, 2008), and the Disease Control Priorities (DCP) third edition chapter on the Organization of Essential Services and the Role of First-Level Hospitals has a strong focus on FRH as a cost-effective platform for surgical care (McCord *et al.*, 2015). WHO also published a guideline on the essential elements of obstetric care at first referral level in 1991 (WHO, 1991), which describes how maternal mortality and morbidity can be addressed at the first referral level; however, a more recent (2016) document on the standards for improving quality of maternal and new-born care in health facilities does not mention FRH (WHO, 2016b).

A similar disappearance of FRH from policy spheres was observed at the level of WHO’s six regional offices, albeit with different approaches.

The Western Pacific Regional Office (WPRO)’s 2019 framework for action on the hospital does not provide guidance on FRH as distinct entities, but rather tackles the hospital sector in an undifferentiated manner (WHO Regional Office for the Western Pacific, 2020). Similarly, the Eastern Mediterranean Regional Office (EMRO) does not address FRH specifically in their 2019 framework for action on hospitals, except to request countries to frame the expected roles and positions for hospital subsectors, which includes FRH among others (WHO Regional Office for the Eastern Mediterranean Region, 2019). In the African Regional Office (AFRO), the absence of FRH was notable from the 2009 Ouagadougou Declaration (WHO Regional Office for Africa, 2010), the 1987 Harare Declaration (WHO, 1987a) and the 2001 Abuja Declaration (Federal Republic of Nigeria, 2001) that discussed PHC and health systems in Africa, strengthening health systems based on PHC and health financing, respectively. Although the Harare Declaration called for the roles and functioning of hospitals to be redefined, a review of its progress in 2013 acknowledged that FRH specifically had fallen off this agenda (Belma, 2013). Despite this, FRH remained absent from subsequent policy documents published in 2016 that attempted to set out a framework for UHC and health financing (The World Bank and WHO, 2016; WHO, 2016a). The South East Asian Regional Office (SEARO), in contrast, appears to have embraced the prevailing discourse on district hospitals in the 1970s, including their role in preventive medicine and the district health approach (Prims, 1970); however, we did not find any recent publication on FRH. Documents from the Regional Office for the Americas (PAHO) also make scant references to FRH, with most references occurring in documents discussing health services following the COVID-19 pandemic (Pan American Health Organization, 2017; 2020). One document talks of the ‘modern general hospital’ with a definition provided that correlates with that of FRH (Pan American Health Organization, 2021), but their role in UHC remains vague (Pan American Health Organization, 2021). In the European Regional Office (EURO), FRH which are referred to as district hospitals, featured prominently in documents related to former Soviet countries (WHO, 2002); however, a 2014 review presented these as a wasteful and ineffective remnant of an archaic Soviet health system (Rechel *et al.*, 2014). More recently in 2021, a Europe-wide health systems priority document omitted FRH from their discussion about redefining hospital care and only discussed hospitals as a homogenous entity (Hansen *et al.*, 2021).

While we did not explore economic literature, within the documents reviewed we did not find any guidance specifically addressing the issue of financing or resource allocation to FRH as distinct service delivery platforms, either at global or at regional levels.

### Scarcity of research and advocacy on FRH in the academic literature

Our review unearthed scarce attention to FRH from the academic community. In 2010, a correspondence was published in *The Lancet*, pleading for investment in district hospitals (Grimes and Lavy, 2010). In 2020, a perspectives paper on the ‘neglected’ district hospitals argued that FRHs are an essential part of the PHC matrix that can provide preventative, curative and diagnostic services that are more cost effective than those offered at higher levels of the health system but are not utilized or supported sufficiently to do so (Roberts *et al.*, 2016; Rajbhandari *et al.*, 2020). In the same year, a correspondence to the International Anaesthesia Research Society was published, lamenting the evidence gap on district-hospital-level anaesthesia and surgery in LMICs: ‘while the academic community continues to publish more and more complex research, the rural district hospitals in low-income countries are being left behind’ (Sund, 2020).

Our review of the research landscape supports this latter point. Our search for academic articles with a synonym for FRH in their title in conjunction with a term for LMICs returned just 132 articles. Excluding opinion pieces, correspondence, commentaries and reviews (*n* = 8), these papers span research on a new tool or intervention (*n* = 46), a population group (*n* = 42), patient outcomes (*n* = 16), hospital staff well-being, knowledge and practices (*n* = 12), costing (*n* = 6) and service accessibility (*n* = 2). In most of these, FRH was not a distinct subject of the research, but rather a study site for the recruitment of patients for epidemiological studies or for trialling a new tool. We identified just seven papers which appear to position FRH as the main subject of their research, among which three relate to surgery.

Regarding the expected clinical services to be available at FRH, only 16 (0.6%) of the 2703 academic articles screened described these, and this was not the central theme in any of these papers.

### Country-level expectations of FRH remain high but funding is unclear

Our fourth and final observation is that the expectations for FRH at the country level remain high, yet it is unclear whether sufficient resources are being provided to achieve these.

We reviewed eight countries’ most recent national health strategic planning documents and found that the majority have included their FRH in some capacity in their planning documents and allocated them a specific role in efforts to improve health outcomes. Nearly all countries have defined service packages for their equivalent of FRH, and half have defined the required staffing norms to support said services. These service packages vary both in categories and content, which we intend to describe in later reports, but the scope of clinical services is universally extensive and undoubtedly both human and material resource-intensive.

Financing patterns for FRH were challenging to untangle, given the tendency in country health financing documents to report on the ‘hospital sector’ at large, sometimes not distinguishing government and out-of-pocket spending and without distinguishing by level of care. Nepal and Sri Lanka for example appear to show divergent patterns; in Nepal, health spending on primary care facilities appears higher than spending on hospitals in the public sector (Government of Nepal, 2018); however, in Sri Lanka, the opposite seems to be true (Rajapaksa *et al.*, 2021). While we could not determine the general trends in the financing of FRH, we observed a high reliance on out-of-pocket payments as a source of hospital funding across the board. This, coupled with the fact that just three of the country case studies (Sri Lanka, Myanmar and Rwanda) explicitly fund FRH services as part of their commitment to UHC, suggests that sufficiently resourcing these facilities likely remains a challenge.

## Discussion

In this review, we have presented a picture of FRH as imagined both historically and currently, and to some extent as implemented in specific countries. Our findings suggest that FRHs are not universally understood, have fallen off the policy agenda and are insufficiently researched. Yet, they continue to exist and are envisaged as playing essential roles within country health systems. This is a concerning set of observations as countries seek to provide a more ambitious scope of services as part of universal health coverage.

Definitions and global guidance matter, as they may set a benchmark for countries to strive towards and, critically, fund. While differences in terminologies are inevitable to some extent and arguably inconsequential, some of the more fundamental aspects of defining “which facilities should provide which services and which of these can be effectively combined in FRH?” would benefit from much greater attention.

The persistent lack of consensus regarding the positioning of FRH ‘within’ PHC vs being ‘complementary’ to it is an important example of this. Such confusion likely impacts on resource allocation to FRH and may even result in the exclusion of FRH from PHC interventions and projects, thus undermining any complementary role of FRH in supporting UHC. This may be further aggravated when specialized professionals seek to promote their own field, which can distort priorities. Strategies to invest in PHC that include FRH may be needed to ensure favourable patient outcomes, as has been argued to reduce maternal and neonatal mortality (WHO, 1991; 2014). This would require clearer guidance in global policy regarding FRH role vis-à-vis PHC.

We suspect that the uncertainty around FRH at least partly explains their disappearance from policy, as distinct service delivery platforms. The recent amalgamation of all hospitals under the ‘hospital sector’ has implications for resource allocation. While running hospitals is expensive and resource-intensive, consuming as much as 50–80% of health budgets in EMRO countries (WHO Regional Office for the Eastern Mediterranean Region, 2019) and 31–56% in WPRO countries (WHO Regional Office for the Western Pacific, 2020), the lack of disaggregated information by hospital level could lead to misinterpretation that FRHs are better financed than they really are (WHO, 2016a). This is concerning, given the observed trend of apportioning considerable hospital sector funding to the construction and functioning of specialist or tertiary facilities (Chabrol *et al.*, 2018). If FRHs are continuously seen as synonymous with specialized health care, large specialist hospitals may consume substantial proportions of health budgets without providing the benefits that FRH offer to large swathes of any countries’ population (Rajbhandari *et al.*, 2020).

There is evidently a need for a greater understanding of how financing is apportioned across levels of the health system and how this might affect FRH. In Nepal, e.g., larger public spending on primary care facilities is likely a result of the large need for such facilities due to the country’s diverse geography, combined with heavy reliance on private hospitals. In Sri Lanka, by contrast, higher spending on hospitals than primary health facilities is explained by lower running costs for the latter, and should not necessarily be interpreted as a skewed resource allocation.

The noted absence of advocates for FRH adds to these concerns. Careful and considered use of terms is warranted by policy makers and academics. Countries already have defined levels of care and, while it would perhaps be unrealistic to attempt to redefine FRH as a unified concept, important country-specific questions remain unanswered, such as which services FRH should and should not provide now and in the medium term. Importantly, FRH should be able to offer these services without compromising on quality of care, which requires a careful and transparent process of defining and prioritizing service packages, ensuring they are appropriately resourced and managed and being clear about how the different levels of care interact with one another to ensure continuity of care (English *et al.*, 2006; Rajbhandari *et al.*, 2020).

While this review did not aim to answer these questions, it seems reasonable for country-level efforts to be made at two levels, as proposed in WHO EMRO’s hospital framework: (1) at the system level through ‘(re) defining their position, roles and functions within national health systems and setting clear objectives for their contribution to universal health coverage and health outcomes’ and (2) at the facility level, through ‘(re)organizing and optimizing hospital production processes to deliver people-centred care and strengthen internal performance to deliver on their mandate’ (WHO Regional Office for the Eastern Mediterranean Region, 2019). Arguably, the latter relies on the former, and the former may be challenging to achieve in the absence of a common understanding of FRH roles, of sufficient resources and of global standards or best practice evidence to guide policy makers in directing resources to the most appropriate level within the health system.

## Conclusion

To conclude, the demand for scarce expertise and essential hospital care provided by FRH in their various forms will continue to exist and fulfil indispensable roles in health systems across the world. FRH will continue to be the nearest available facilities offering life-saving care for acute severe illness and services that address increasingly complex health issues to swathes of populations in LMICs, especially in countries where physicians are rarely found in public, primary care facilities. In the era of sustainable development goals, DCP3 proposes that countries could aim ‘to ensure most patients have access to fully resourced, high-quality, first-level hospitals—a goal that, although aspirational, could be feasible by 2030’ (Watkins *et al.*, 2017). While our review has not provided answers on how to achieve this, it highlights the need for the global health community to recognize the importance of FRH as a shared service delivery platform and reconsider how they can be strengthened in an integrated, health system strengthening approach.

## Supplementary Material

czad120_Supp

## Data Availability

No primary data were collected for this review. The search strategies are made available in appendices and further information on the literature can be provided by the authors.

## Reflexivity statement

This work was undertaken by a group of researchers based in both high- and low-resource countries across Africa, Asia and Europe. The author group comprises four females and two males, with varying research seniority levels and academic backgrounds and qualifications,. The lead researchers were two female junior researchers who drafted this paper with the guidance of Professor M.E., an Oxford-based researcher with decades of experience in East Africa. The authors from Burundi, Myanmar and Nepal provided perspective from countries with different health systems to those experienced by the lead researchers, which vastly strengthened the research and provided opportunities to consider how individual countries fit within a global picture.
