# Supplementary material for: First referral hospitals in low- and middle-income countries: the need for a renewed focus
Source: Health Policy Plan. 2023 Dec 20;39(2):224–32. doi: 10.1093/heapol/czad120 (PMC11031140; doi:10.1093/heapol/czad120)
Supplement: czad120_Supp [file czad120_supp.zip › suppl_data/FLH policy review_supplementary material 1 to 3_HPP.docx]

**Supplementary Material 1: Search strategy and selection criteria- structured academic literature review**

| **Step 1: Review of Lancet commissions**   - All commissions published by the Lancet(The Lancet, 2022a) (n=85) were purposively title and summary screened by two reviewers, for relevance/inclusions (using Rayyan). All conflicts were discussed and addressed. - Articles selected for full text review were divided between two reviewers - Included articles were divided between two reviewers for data extraction into an excel-based tool - Both reviewers cross checked the extracted data to ensure standardised approach and inclusion validity - Articles where there was uncertainty or lack of consensus were shared with a senior reviewer and underwent a second round of exclusions accordingly to derive a final list of included articles.   Articles title and abstract /summary screened (n=85)  Articles excluded  (n= 54)  Articles full text reviewed (n = 31)  Articles excluded in Round 1 (n=6)  Articles re-assessed for inclusion in second review (n = 25)  Articles excluded in Round 2 (n=20)  Articles included in final review  (n = 5)  **Step 2: Review of Lancet series**   - All series articles published by the Lancet(The Lancet, 2022b) (n= 1922) were divided between two reviewers for title and summary screening, directly through the Lancet series website - Articles selected for full text review were divided between two reviewers - Included articles were divided between two reviewers for data extraction into an excel-based tool - Both reviewers cross checked the extracted data to ensure standardised approach and inclusion validity - Articles where there was uncertainty or lack of consensus were shared with a senior reviewer and underwent a second round of exclusions accordingly, to derive a final list of included articles.   Articles title and abstract /summary screened (n=1922)  Articles excluded  (n= 1876)  Articles excluded in Round 1 (n=28)  Articles full text reviewed (n = 46)  Articles excluded in Round 2 (n=10)  Articles re-assessed for inclusion in second review (n = 18)  Articles included in final review  (n = 8)  **Step 3: structured literature review on FRH expected clinical services**   - An information specialist (Elinor Harriss) searched the following databases on 03/08/2022 with no limits for language or publication dates: Ovid Embase; Ovid Medline; Ovid Global Health; Ovid PsycINFO; Ovid AMED; EBSCOhost CINAHL; the Cochrane Database of Systematic Reviews; the Cochrane Central Register of Controlled Trials. - The search strategies used text words and relevant indexing to retrieve relevant literature, incorporating an adapted version of the EPOC General LMIC terms for Ovid (Cochrane Effective Practice and Organisation of Care, 2020) based on the search strategy indicated in Supplementary Material 2 - All references were exported to Endnote 20 (Thomson Reuters, New York, NY), and duplicates were removed using the Deduklick programme developed by Risklick. The reference lists of included papers were assessed for additional relevant studies. - All references were title and abstract screened by two reviewers, using Rayyan - Full text review was done by one reviewer, to identify papers for inclusion - Additional articles from a pre-existing repository of FRH articles created by a senior researcher were also included during full text review. - Included articles were split between two reviewers for data extraction into an excel-based tool - Both reviewers cross checked the extracted data to ensure standardised approach and inclusion validity - Articles where there was uncertainty or lack of consensus were shared with a senior reviewer and underwent a second round of exclusions accordingly, to derive a final list of included articles   Articles excluded (n=641)  Articles title and abstract /summary screened from search strategy (n=680)  Articles from search strategy text reviewed (n =39)  Articles from pre-existing repository full text reviewed (n=16)  Articles excluded in Round 1 (n= 34)  Articles excluded in Round 1 (n= 13)  Articles excluded in Round 2 (n= 4)  Articles from search strategy re-assessed for inclusion in second review (n = 5)  Articles from pre-existing repository re-assessed for inclusion in second review (n=3)  Articles excluded in Round 2 (n= 1)  Articles included in final review from search strategy (n=1)  Articles included in final review from pre-existing repository (n=2)  (n = 3)  *Note: for steps 1-3, our interest was to understand what clinical services are expected of FRH. Thus, we included academic articles that provide recommendations of health services expected to be delivered by FRH (or an equivalent synonym) that are either global or LMIC region focused. We excluded articles that specifically addressed high income settings, or are country-focused, or provided recommendations that fall outside of the scope of health services or provided health service recommendations without specifying the service delivery platform. Given that our interest and focus in this review was on services, we complemented our review with Step 4, as described below.*  **Step 4: Structured scoping of FRH themed papers**   - We searched the following databases on 10/02/2023 with no limits for language or publication dates: Ovid Embase; Ovid Medline; Ovid Global Health; Ovid PsycINFO; Ovid AMED. - The search strategies used text words and relevant indexing to retrieve relevant literature, based on the search strategy indicated in Supplementary Material 3 - All references were exported to Rayyan and duplicates were removed using Rayyan - To characterise the research landscape on FRH, references were title and abstract screened by two reviewers and categorised thematically based on the type of research paper; in excel - Both reviewers cross checked the categorisation to ensure standardised approach |
| --- |

**Supplementary Material 2: Search Strategy for FRH expected clinical services**

--------------------------------------------------------------------------------

Database: Embase 1974 to present

1 (developing country or developing countries or developing nation? or developing population? or developing world or less developed countr* or less developed nation? or less developed population? or less developed world or lesser developed countr* or lesser developed nation? or lesser developed population? or lesser developed world or under developed countr* or under developed nation? or under developed population? or under developed world or underdeveloped countr* or underdeveloped nation? or underdeveloped population? or underdeveloped world or middle income countr* or middle income nation? or middle income population? or low income countr* or low income nation? or low income population? or lower income countr* or lower income nation? or lower income population? or underserved countr* or underserved nation? or underserved population? or underserved world or under served countr* or under served nation? or under served population? or under served world or deprived countr* or deprived nation? or deprived population? or deprived world or poor countr* or poor nation? or poor population? or poor world or poorer countr* or poorer nation? or poorer population? or poorer world or developing econom* or less developed econom* or lesser developed econom* or under developed econom* or underdeveloped econom* or middle income econom* or low income econom* or lower income econom* or low gdp or low gnp or low gross domestic or low gross national or lower gdp or lower gnp or lower gross domestic or lower gross national or lmic or lmics or third world or lami countr* or transitional countr* or global south or emerging economies or emerging nation? or AFRO or EMRO or SEARO or WPRO).ti,ab,kw. (169474)

2 exp Africa/ or (africa or magreb or maghrib).mp. (423251)

3 (asia or subcontinent or sub-continent).mp. or Asia/ (162846)

4 caribbean.mp. or Caribbean/ or west indies.mp. or Caribbean Islands/ (24395)

5 (south america or "pacific region").mp. or South America/ or "South and Central America"/ (51570)

6 1 or 2 or 3 or 4 or 5 (740216)

7 exp policy/ (315931)

8 (Policy or policies or SOP or Guidance or Guideline* or Protocol* or Strategy or report*).ti,ab,kw. (7785877)

9 practice guideline/ (506931)

10 7 or 8 or 9 (8120305)

11 ("general hospital*" or "county hospital*" or (District* adj2 hospital*) or (Level 1 adj1 hospital*) or (Primary* adj2 hospital*) or (Community adj1 hospital*) or (Rural adj1 hospital*) or (County adj1 hospital*) or (First adj2 hospital*)).ti,ab,kw. (135229)

12 (health* adj1 service*).ti,ab,kw. (166852)

13 (hospital* adj1 service*).ti,ab,kw. (9974)

14 12 or 13 (174845)

15 6 and 10 and 11 and 14 (242)

--------------------------------------------------------------------------------

Database: Medline (Ovid MEDLINE® Epub Ahead of Print, In-Process & Other Non-Indexed Citations, Ovid MEDLINE® Daily and Ovid MEDLINE®) 1946 to present

1 (developing country or developing countries or developing nation? or developing population? or developing world or less developed countr* or less developed nation? or less developed population? or less developed world or lesser developed countr* or lesser developed nation? or lesser developed population? or lesser developed world or under developed countr* or under developed nation? or under developed population? or under developed world or underdeveloped countr* or underdeveloped nation? or underdeveloped population? or underdeveloped world or middle income countr* or middle income nation? or middle income population? or low income countr* or low income nation? or low income population? or lower income countr* or lower income nation? or lower income population? or underserved countr* or underserved nation? or underserved population? or underserved world or under served countr* or under served nation? or under served population? or under served world or deprived countr* or deprived nation? or deprived population? or deprived world or poor countr* or poor nation? or poor population? or poor world or poorer countr* or poorer nation? or poorer population? or poorer world or developing econom* or less developed econom* or lesser developed econom* or under developed econom* or underdeveloped econom* or middle income econom* or low income econom* or lower income econom* or low gdp or low gnp or low gross domestic or low gross national or lower gdp or lower gnp or lower gross domestic or lower gross national or lmic or lmics or third world or lami countr* or transitional countr* or global south or emerging economies or emerging nation? or AFRO or EMRO or SEARO or WPRO).ti,ab,kw. (161168)

2 exp Africa/ or (africa or magreb or maghrib).mp. (361071)

3 (asia or subcontinent or sub-continent).mp. or Asia/ (108435)

4 caribbean.mp. or Caribbean Region/ or west indies.mp. or West Indies/ (21863)

5 (south america or "pacific region").mp. or South America/ or Central America/ (32891)

6 1 or 2 or 3 or 4 or 5 (593239)

7 exp policy/ (171473)

8 (Policy or policies or SOP or Guidance or Guideline* or Protocol* or Strategy or report*).ti,ab,kw. (5990391)

9 exp guideline/ (37174)

10 7 or 8 or 9 (6093342)

11 ("general hospital*" or "county hospital*" or (District* adj2 hospital*) or (Level 1 adj1 hospital*) or (Primary* adj2 hospital*) or (Community adj1 hospital*) or (Rural adj1 hospital*) or (County adj1 hospital*) or (First adj2 hospital*)).ti,ab,kw. (88609)

12 (health* adj1 service*).ti,ab,kw. (139166)

13 (hospital* adj1 service*).ti,ab,kw. (7421)

14 12 or 13 (144997)

15 6 and 10 and 11 and 14 (224)

--------------------------------------------------------------------------------

Database: Global Health <1973 to 2022 Week 30>

1 (developing country or developing countries or developing nation? or developing population? or developing world or less developed countr* or less developed nation? or less developed population? or less developed world or lesser developed countr* or lesser developed nation? or lesser developed population? or lesser developed world or under developed countr* or under developed nation? or under developed population? or under developed world or underdeveloped countr* or underdeveloped nation? or underdeveloped population? or underdeveloped world or middle income countr* or middle income nation? or middle income population? or low income countr* or low income nation? or low income population? or lower income countr* or lower income nation? or lower income population? or underserved countr* or underserved nation? or underserved population? or underserved world or under served countr* or under served nation? or under served population? or under served world or deprived countr* or deprived nation? or deprived population? or deprived world or poor countr* or poor nation? or poor population? or poor world or poorer countr* or poorer nation? or poorer population? or poorer world or developing econom* or less developed econom* or lesser developed econom* or under developed econom* or underdeveloped econom* or middle income econom* or low income econom* or lower income econom* or low gdp or low gnp or low gross domestic or low gross national or lower gdp or lower gnp or lower gross domestic or lower gross national or lmic or lmics or third world or lami countr* or transitional countr* or global south or emerging economies or emerging nation? or AFRO or EMRO or SEARO or WPRO).mp. (1035377)

2 exp Africa/ or (africa or magreb or maghrib).mp. (264851)

3 (asia or subcontinent or sub-continent).mp. or Asia/ (729187)

4 caribbean.mp. or Caribbean/ or west indies.mp. (21274)

5 (south america or "pacific region").mp. or South America/ or Central America/ (163189)

6 1 or 2 or 3 or 4 or 5 (1226687)

7 exp policy/ (56775)

8 (Policy or policies or SOP or Guidance or Guideline* or Protocol* or Strategy or report*).ti,ab. (923394)

9 guidelines/ (61529)

10 7 or 8 or 9 (946085)

11 ("general hospital*" or "county hospital*" or (District* adj2 hospital*) or (Level 1 adj1 hospital*) or (Primary* adj2 hospital*) or (Community adj1 hospital*) or (Rural adj1 hospital*) or (County adj1 hospital*) or (First adj2 hospital*)).mp. (18637)

12 (health* adj1 service*).ti,ab. (40285)

13 (hospital* adj1 service*).ti,ab. (1365)

14 12 or 13 (41282)

15 6 and 10 and 11 and 14 (292)

--------------------------------------------------------------------------------

Database: PsycINFO 1806 to present

1 (developing country or developing countries or developing nation? or developing population? or developing world or less developed countr* or less developed nation? or less developed population? or less developed world or lesser developed countr* or lesser developed nation? or lesser developed population? or lesser developed world or under developed countr* or under developed nation? or under developed population? or under developed world or underdeveloped countr* or underdeveloped nation? or underdeveloped population? or underdeveloped world or middle income countr* or middle income nation? or middle income population? or low income countr* or low income nation? or low income population? or lower income countr* or lower income nation? or lower income population? or underserved countr* or underserved nation? or underserved population? or underserved world or under served countr* or under served nation? or under served population? or under served world or deprived countr* or deprived nation? or deprived population? or deprived world or poor countr* or poor nation? or poor population? or poor world or poorer countr* or poorer nation? or poorer population? or poorer world or developing econom* or less developed econom* or lesser developed econom* or under developed econom* or underdeveloped econom* or middle income econom* or low income econom* or lower income econom* or low gdp or low gnp or low gross domestic or low gross national or lower gdp or lower gnp or lower gross domestic or lower gross national or lmic or lmics or third world or lami countr* or transitional countr* or global south or emerging economies or emerging nation? or AFRO or EMRO or SEARO or WPRO).mp. (29997)

2 exp Africa/ or (africa or magreb or maghrib).mp. (28094)

3 (asia or subcontinent or sub-continent).mp. or Asia/ (11321)

4 caribbean.mp. or Caribbean/ or west indies.mp. (4767)

5 (south america or "pacific region").mp. or South America/ or Central America/ (2422)

6 1 or 2 or 3 or 4 or 5 (68483)

7 exp Health Care Policy/ or exp Policy Making/ (92625)

8 (Policy or policies or SOP or Guidance or Guideline* or Protocol* or Strategy or report*).ti,ab. (1178674)

9 7 or 8 (1210528)

10 ("general hospital*" or "county hospital*" or (District* adj2 hospital*) or (Level 1 adj1 hospital*) or (Primary* adj2 hospital*) or (Community adj1 hospital*) or (Rural adj1 hospital*) or (County adj1 hospital*) or (First adj2 hospital*)).mp. (12201)

11 (health* adj1 service*).ti,ab. (55476)

12 (hospital* adj1 service*).ti,ab. (1356)

13 11 or 12 (56508)

14 6 and 9 and 10 and 13 (22)

--------------------------------------------------------------------------------

Database: AMED (Allied and Complementary Medicine) <1985 to July 2022>

1 (developing country or developing countries or developing nation? or developing population? or developing world or less developed countr* or less developed nation? or less developed population? or less developed world or lesser developed countr* or lesser developed nation? or lesser developed population? or lesser developed world or under developed countr* or under developed nation? or under developed population? or under developed world or underdeveloped countr* or underdeveloped nation? or underdeveloped population? or underdeveloped world or middle income countr* or middle income nation? or middle income population? or low income countr* or low income nation? or low income population? or lower income countr* or lower income nation? or lower income population? or underserved countr* or underserved nation? or underserved population? or underserved world or under served countr* or under served nation? or under served population? or under served world or deprived countr* or deprived nation? or deprived population? or deprived world or poor countr* or poor nation? or poor population? or poor world or poorer countr* or poorer nation? or poorer population? or poorer world or developing econom* or less developed econom* or lesser developed econom* or under developed econom* or underdeveloped econom* or middle income econom* or low income econom* or lower income econom* or low gdp or low gnp or low gross domestic or low gross national or lower gdp or lower gnp or lower gross domestic or lower gross national or lmic or lmics or third world or lami countr* or transitional countr* or global south or emerging economies or emerging nation? or AFRO or EMRO or SEARO or WPRO).mp. (973)

2 exp Africa/ or (africa or magreb or maghrib).mp. (2249)

3 (asia or subcontinent or sub-continent).mp. or Asia/ (2172)

4 (caribbean or "west indies").mp. (106)

5 (south america or "pacific region").mp. or South America/ (740)

6 1 or 2 or 3 or 4 or 5 (5913)

7 health policy/ (1264)

8 (Policy or policies or SOP or Guidance or Guideline* or Protocol* or Strategy or report*).mp. (64157)

9 exp guidelines/ (2602)

10 7 or 8 or 9 (64157)

11 ("general hospital*" or "county hospital*" or (District* adj2 hospital*) or (Level 1 adj1 hospital*) or (Primary* adj2 hospital*) or (Community adj1 hospital*) or (Rural adj1 hospital*) or (County adj1 hospital*) or (First adj2 hospital*)).mp. (1189)

12 (health* adj1 service*).mp. (11350)

13 (hospital* adj1 service*).mp. (154)

14 12 or 13 (11447)

15 6 and 10 and 11 and 14 (2)

# Query Results

S1 TX developing country or developing countries or developing nation? or developing population? or developing world or less developed countr* or less developed nation? or less developed population? or less developed world or lesser developed countr* or lesser developed nation? or lesser developed population? or lesser developed world or under developed countr* or under developed nation? or under developed population? or under developed world or underdeveloped countr* or underdeveloped nation? or underdeveloped population? or underdeveloped world or middle income countr* or middle income nation? or middle income population? or low income countr* or low income nation? or low income population? or lower income countr* or lower income nation? or lower income population? or underserved countr* or underserved nation? or underserved population? or underserved world or under served countr* or under served nation? or under served population? or under served world or deprived countr* or deprived nation? or deprived population? or deprived world or poor countr* or poor nation? or poor population? or poor world or poorer countr* or poorer nation? or poorer population? or poorer world or developing econom* or less developed econom* or lesser developed econom* or under developed econom* or underdeveloped econom* or middle income econom* or low income econom* or lower income econom* or low gdp or low gnp or low gross domestic or low gross national or lower gdp or lower gnp or lower gross domestic or lower gross national or lmic or lmics or third world or lami countr* or transitional countr* or global south or emerging economies or emerging nation? or AFRO or EMRO or SEARO or WPRO 66,745

S2 (MH "Africa+") 95,234

S3 TX africa or magreb or maghrib 79,523

S4 (MH "Asia+") 330,259

S5 TX asia or subcontinent or sub-continent 209,770

S6 (MH "West Indies+") 11,122

S7 TX caribbean OR "west indies" 8,069

S8 (MH "Central America+") OR (MH "South America+") 72,150

S9 TX "south america" or "pacific region" 59,262

S10 S1 OR S2 OR S3 OR S4 OR S5 OR S6 OR S7 OR S8 OR S9 741,832

S11 (MH "Health Policy+") OR (MH "Organizational Policies+") OR (MH "Hospital Policies+") OR (MH "Policy Making") 129,728

S12 (MH "Practice Guidelines") 84,876

S13 TI ( Policy or policies or SOP or Guidance or Guideline* or Protocol* or Strategy or report* ) OR AB ( Policy or policies or SOP or Guidance or Guideline* or Protocol* or Strategy or report* ) 1,592,718

S14 S11 OR S12 OR S13 1,701,700

S15 TI ("general hospital*" or "county hospital*" or (District* n2 hospital*) or ("Level 1" n1 hospital*) or (Primary* n2 hospital*) or (Community n1 hospital*) or (Rural n1 hospital*) or (County n1 hospital*) or (First n2 hospital*)) OR AB ("general hospital*" or "county hospital*" or (District* n2 hospital*) or ("Level 1" n1 hospital*) or (Primary* n2 hospital*) or (Community n1 hospital*) or (Rural n1 hospital*) or (County n1 hospital*) or (First n2 hospital*)) 38,647

S16 TI health* n1 service* OR AB health* n1 service* 97,027

S17 TI hospital* n1 service* OR AB hospital* n1 service* 6,070

S18 S16 OR S17 101,517

S19 S10 AND S14 AND S15 AND S18 298

--------------------------------------------------------------------------------

Cochrane Database of Systematic Reviews

Issue 8 of 12, August 2022

Cochrane Central Register of Controlled Trials

Issue 7 of 12, July 2022

#1 ("developing country" or "developing countries" or "developing nation*" or "developing population*" or "developing world" or "less developed countr*" or "less developed nation*" or "less developed population*" or "less developed world" or "lesser developed countr*" or "lesser developed nation*" or "lesser developed population*" or "lesser developed world" or "under developed countr*" or "under developed nation*" or "under developed population*" or "under developed world" or "underdeveloped countr*" or "underdeveloped nation*" or "underdeveloped population*" or "underdeveloped world" or "middle income countr*" or "middle income nation*" or "middle income population*" or "low income countr*" or "low income nation*" or "low income population*" or "lower income countr*" or "lower income nation*" or "lower income population*" or "underserved countr*" or "underserved nation*" or "underserved population*" or "underserved world" or "under served countr*" or "under served nation*" or "under served population*" or "under served world" or "deprived countr*" or "deprived nation*" or "deprived population*" or "deprived world" or "poor countr*" or "poor nation*" or "poor population*" or "poor world" or "poorer countr*" or "poorer nation*" or "poorer population*" or "poorer world" or "developing econom*" or "less developed econom*" or "lesser developed econom*" or "under developed econom*" or "underdeveloped econom*" or "middle income econom*" or "low income econom*" or "lower income econom*" or "low gdp" or "low gnp" or "low gross domestic" or "low gross national" or "lower gdp" or "lower gnp" or "lower gross domestic" or "lower gross national" or lmic or lmics or "third world" or "lami countr*" or "transitional countr*" or "global south" or "emerging economies" or "emerging nation*" or AFRO or EMRO or SEARO or WPRO):ti,ab,kw 5606

#2 (africa or magreb or maghrib):ti,ab,kw 7899

#3 (asia or subcontinent or sub-continent):ti,ab,kw 5285

#4 (caribbean or "west indies"):ti,ab,kw 631

#5 ("south america" or "pacific region"):ti,ab,kw 913

#6 #1 or #2 or #3 or #4 or #5 18318

#7 (Policy or policies or SOP or Guidance or Guideline* or Protocol* or Strategy or report*):ti,ab,kw 450849

#8 ("general hospital*" or "county hospital*" or (District* near/2 hospital*) or ("Level 1" near/1 hospital*) or (Primary* near/2 hospital*) or (Community near/1 hospital*) or (Rural near/1 hospital*) or (County near/1 hospital*) or (First near/2 hospital*)):ti,ab,kw 9008

#9 ((health* OR hospital*) near/1 service*):ti,ab,kw 25894

#10 #6 and #7 and #8 and #9 18

**Supplementary Material 3: Search strategy for scoping of FRH themed papers**

Database: AMED (Allied and Complementary Medicine) <1985 to January 2023>, Embase 1974 to present, Global Health <1973

to 2023 Week 06>, Medline (Ovid MEDLINE® Epub Ahead of Print, In-Process & Other Non-Indexed Citations, Ovid MEDLINE®

Daily and Ovid MEDLINE®) 1946 to present, PsycINFO 1806 to present

1 hospital at first referral level.ti. (0)

2 first referral hospital.ti. (3)

3 first referral level hospital.ti. (0)

4 1 or 2 or 3 (3)

5 remove duplicates from 4 (2)

6 district hospital.ti. (3636)

7 (developing country or developing countries or developing nation? or developing population? or developing world or

less developed countr* or less developed nation? or less developed population? or less developed world or lesser

developed countr* or lesser developed nation? or lesser developed population? or lesser developed world or under

developed countr* or under developed nation? or under developed population? or under developed world or underdeveloped

countr* or underdeveloped nation? or underdeveloped population? or underdeveloped world or middle income countr* or

middle income nation? or middle income population? or low income countr* or low income nation? or low income population?

or lower income countr* or lower income nation? or lower income population? or underserved countr* or underserved

nation? or underserved population? or underserved world or under served countr* or under served nation? or under served

population? or under served world or deprived countr* or deprived nation? or deprived population? or deprived world or

poor countr* or poor nation? or poor population? or poor world or poorer countr* or poorer nation? or poorer population?

or poorer world or developing econom* or less developed econom* or lesser developed econom* or under developed econom*

or underdeveloped econom* or middle income econom* or low income econom* or lower income econom* or low gdp or low gnp

or low gross domestic or low gross national or lower gdp or lower gnp or lower gross domestic or lower gross national or

lmic or lmics or third world or lami countr* or transitional countr* or global south or emerging economies or emerging

nation? or AFRO or EMRO or SEARO or WPRO).ti,kw,ab. (446942)

8 6 and 7 (254)

9 remove duplicates from 8 (143)

10 5 or 9 (145)

11 remove duplicates from 10 (133)
