# Supplementary material for: First referral hospitals in low- and middle-income countries: the need for a renewed focus
Source: Health Policy Plan. 2023 Dec 20;39(2):224–32. doi: 10.1093/heapol/czad120 (PMC11031140; doi:10.1093/heapol/czad120)
Supplement: czad120_Supp [file czad120_supp.zip › suppl_data/FLH policy review_supplementary material 4 to 6_HPP.docx]

**Supplementary Material 4: Country case study data sources**

| Countries | Government documents | Other sources |
| --- | --- | --- |
| Vietnam | 1. The Ministry of Health of Viet Nam. Medical Services Administration [Internet]. 2018 [cited 2022 Sep 13]. Available from: <http://hoinhap.kcb.vn/en/gioi-thieu/> 2. Ministry of Health of Viet Nam. Lessons for Hospital Autonomy Implementation in Vietnam from International Experience Issues identified from International Studies and a Public Hospital Survey in Vietnam The World Bank Vietnam Office. 2011. 3. Ministry of Health. Health Statistics Yearbook 2018 [Internet]. [cited 2022 Oct 26]. Available from: https://moh.gov.vn/thong-ke-y-te/-/asset_publisher/nEY3Q7enxRKG/content/nien-giam-thong-ke-y-te-nam-2018 4. Ministry of Health of Vietnam HPG. Joint Annual Health Review JAHR 2015: Strengthening Primary Health Care At The Grassroots Towards Universal Health Coverage”. . In 2015. 5. Viet Nam Government. National Design Standard for District Hospitals in Viet Nam . 2012. | 1. World Bank. World Bank: Data for Viet Nam [Internet]. 2022 [cited 2022 Sep 12]. Available from: https://data.worldbank.org/?locations=VN-XN 2. Vietnam.Tổng cục thống kê. Niên giám thống kê = Statistical yearbook of Viet Nam 2020. 1055 p. 3. Viet Nam Law. Current local administration system in Viet Nam [Internet]. [cited 2022 Sep 12]. Available from: <https://vietnamlawmagazine.vn/current-local-administration-system-in-vietnam-6058.html> 4. Ho TVT, Cottrell A, Valentine P, Woodley S. Perceived barriers to effective multilevel governance of human-natural systems: An analysis of marine protected areas in Vietnam. J Polit Ecol. 2012;19(1):17–35 5. Thi Mai Oanh T, Khanh Phuong N, Anh Tuan K. Sustainability and Resilience in the Vietnamese Health System Sustainability and Resilience in the Vietnamese Health System Sustainability and Resilience in the Vietnamese Health System [Internet]. 2021. Available from: https://weforum.org/phssr 6. Ellner AL, Duong DB, Giesen L, Ibrahim Z, Khan K, Bitton A, et al. Primary Health Care in Vietnam-Assessing the Doi Moi Reforms and Building a Vision for the Future. 7. Hoa NT. Primary care in Central Vietnam: Measurement, Assessment and Perception of users and providers. 2021. 8. WHO Western Pacific Region. Human Resources for Health Country Profiles - Viet Nam. 2016. 9. WHO. Health Financing [Internet]. [cited 2022 Oct 23]. Available from: https://www.who.int/vietnam/health-topics/health-financing 10. WHO. Global Health Expenditure Database (GHED) [Internet]. [cited 2022 Sep 15]. Available from: https://apps.who.int/nha/database/ViewData/Indicators/en 11. The World Bank. Current health expenditure (% of GDP) [Internet]. [cited 2022 Sep 15]. Available from: https://data.worldbank.org/indicator/SH.XPD.CHEX.GD.ZS?locations=VN 12. Le DC, Kubo T, Fujino Y, Pham TM, Matsuda S. Health Care System in Vietnam: Current Situation and Challenges. Asian Pacific Journal of Disease Management. 2010;4(2):23–30. 13. Le QN, Blizzard L, Si L, Giang LT, Neil AL. The evolution of social health insurance in Vietnam and its role towards achieving universal health coverage. Health Policy Open. 2020 Dec 1;1. 14. The World Bank. The World Bank. Physicians (per 1,000 people). (n.d.). [Internet]. [cited 2022 Oct 26]. Available from: <https://data.worldbank.org/indicator/SH.MED.PHYS.ZS?locations=VN> 15. Tran TD, Vu PM, Pham HTM, Au LN, Do HP, Doan HTT, et al. Transforming medical education to strengthen the health professional training in Viet Nam: A case study. Lancet Reg Health West Pac. 2022 Oct;27:100543. 16. World Health Organization. DISTRICT HOSPITALS: GUIDELINES FOR DEVELOPMENT Second Edition. 1996. |
| South Africa | Department of Health. Annual Performance Plan 2021/2022. Accessed 25th Aug 2022. Available at: [Annual Performance Plan – National Department of Health](https://www.health.gov.za/annual-performance-plan/)  Government Gazette. National Health Amendment Act No.12 of 2013. 2013. Accessed 6^th^ Aug 2022. Available at: [National Health Amendment Act (www.gov.za)](https://www.gov.za/sites/default/files/gcis_document/201409/36702gon529_1.pdf)  National Treasury. People’s guide #RSABUDGET2019. 2019. Accessed on 6h Sept 2022. Available at: [2019 Peoples Guide English.pdf (treasury.gov.za)](http://www.treasury.gov.za/documents/national%20budget/2019/guides/2019%20Peoples%20Guide%20English.pdf)  USER GUIDE - UPFS 2022: Uniform User Fee Schedule Policy Full Paying Users Attending Public Hospitals. 2022. Accessed 31th Aug 2022. Available at: [69921_UPFS USER GUIDE 2022_EDITED OCTOBER 2021_NEW (004).doc (live.com)](https://view.officeapps.live.com/op/view.aspx?src=https%3A%2F%2Fhms2.southafricanorth.cloudapp.azure.com%2Fhms2%2Fdocuments%2F69921_UPFS%2520USER%2520GUIDE%25202022_EDITED%2520OCTOBER%25202021_NEW%2520(004).doc&wdOrigin=BROWSELINK)  National Health Act. Regulations relating to categories of hospitals-updated version. 2012. Accessed 23^rd^ Aug 2022. Available at: [rrtcoh462.pdf (saflii.org)](http://www.saflii.org/za/legis/consol_reg/rrtcoh462.pdf)  Department of Health, White Paper for the Transformation of the Health System in South Africa, Government Gazette, # 17910. 1997, National Department of Health: Pretoria. Available online: http://www.doh.gov.za/docs/policy/white_paper/healthsys97_01ht  National Department of Health. Annual Report 2020/2021. 2021. Accessed 5^th^ Sept 2022. Available at: Department of Health Annual Report 2020/2021 (www.gov.za)  KZN Department of Health. District Health System: Situational Analysis. 2004. Accessed 1^st^ Sept 2022. Available at: [Microsoft Word - 5. Anex 2 - DHS update 26042005.doc (kznhealth.gov.za)](https://www.kznhealth.gov.za/strat/annex2.pdf) | 1. UNICEF. South Africa (ZAF) - Demographics, Health & Infant Mortality - UNICEF DATA. Accessed 12^th^ Aug 2022. Available at: [South Africa (ZAF) - Demographics, Health & Infant Mortality - UNICEF DATA](https://data.unicef.org/country/zaf/)   Dell AJ, Kahn D. Geographical maldistribution of surgical resources in South Africa: A review of the number of hospitals, hospital beds and surgical beds. **South African Medical Journal**, [S.l.], v. 107, n. 12, p. 1099-1105, nov. 2017. ISSN 2078-5135. doi:10.7196/SAMJ.2017.v107i12.12539.  Maphumulo WT, Bhengu BR. Challenges of quality improvement in the healthcare of South Africa post-apartheid: A critical review. Curationis. 2019;42(1):1-9.  Ruud KW, Srinivas SC, Toverud EL. Antiretroviral therapy in a South African public health care setting – facilitating and constraining factors. *Southern Med Review* (2009) 2; 2:29–34  McIntyre D, Bloom G, Doherty J, Brijlal P. Health expenditure and finance in South Africa. Durban: Health Systems Trust and the World Bank, 1995  A Rajman & OH Mahomed (2019) Prevalence and determinants of self-directed referrals amongst patients at hospitals in eThekwini District, KwaZulu-Natal 2015, South African Family Practice, 61:2, 53-59, DOI: [10.1080/20786190.2019.1582213](https://doi.org/10.1080/20786190.2019.1582213)  Campbell J, Dussault G, Buchan J, Pozo-Martin F, Guerra Arias M, Leone C,et al. A universal truth: no health without a workforce. Geneva: WorldHealth Organization; 2013.  Labonté R, Sanders D, Mathole, T, Crush, J, Chikanda A, Dambisya Y., Runnels, V., Packer, C., Mackenzie, A., Murphy, G.T., Bourgeault, I.L., 2015. Health worker migration from South Africa: causes, consequences and policy responses. Human Resources for Health 13.. doi:10.1186/s12960-015-0093-4  Stewart J, Wolvaardt G. Hospital management and health policy—a South African perspective*. J Hosp Manag Health Policy* 2019;3:14.  Govender I, Steyn C, Maphasha O, Abdulrazak, A., 2019. A profile of Caesarean sections performed at a district hospital in Tshwane, South Africa. South African Family Practice 61, 246–251. doi:10.1080/20786190.2019.1671655 |
| Rwanda | 1. Government of Rwanda: Administrative structure 2022 [Available from: <https://www.gov.rw/government/administrative-structure>. 2. Ministry of Health: Fourth Health Sector Strategic Plan 2018-2024. In: Health Mo, editor. Kigali: Government of Rwanda; 2018. p. 4. 3. (NISR) NIoSoR. Child Health and Nutrition: Rwanda Demographic and Health Survey (RDHS) Key Findings [Online]. Rwanda Public Health Bulletin. 2021;3(1):19-21. 4. Ministry of Health. National Community Health Strategic Plan 2013-2018 In: Health Mo, editor. Kigali: Government of Rwanda; 2013. p. 2. 5. Ministry of Health. Fourth Health Sector Strategic Plan:  Annex 6 Service Package interventions by level. In: Health Mo, editor. Kigali: Government of Rwanda; 2018. p. 11(Annex). 6. Ministry of Health. Health Financing Strategic Plan 2018-2024. In: Health Mo, editor. Kigali: Government of Rwanda; 2019. 7. Official Rwanda Development Board (RDB) Website 2022 [Available from: <https://rdb.rw/investment-opportunities/health-services/>. 8. Ministry of Health. Health Service Packages  for Public Health Facilities: Annex A-E. In: Health Mo, editor. Kigali: Government of Rwanda; 2017. 9. Ministry of Health. Health Service Packages  for Public Health Facilities. In: Health Mo, editor. Kigali: Government of Rwanda; 2017. p. 113-7. 10. Ministry of Health. Integrated National Health Sector Referral  Guidelines (INHSRG)  In: Health Mo, editor. Kigali2020. p. 9. 11. Ministry of Health. Health Sector Policy. In: Health Mo, editor. Online: Government of Rwanda; 2005. 12. Ministry of Health. 10-Year Government Program:  National Strategy For Health Professions Development  2020 – 2030  Kigali: Government of Rwanda. 13. Ministry of Health. Integrated National Health Sector Referral  Guidelines (INHSRG)  In: Health Mo, editor. Kigali: Government of Rwanda; 2020. p. 11. 14. Ministry of Health. Health Service Packages for Public Health Facilities. In: Health Mo, editor. Kigali2017. p. 9. 15. National Institute of Statistics (NIS) [Rwanda] MoHM, Inc RaMI. Rwanda Service Provision Assessment Survey (2007). In: (MOH) MoH, editor. Calverton, MD, USA: NIS, MOH and Macro International Inc; 2008. | 1. Bank W. Rwanda Overview: Development news, research, data \| World Bank: World Bank; 2022 [Available from: <https://www.worldbank.org/en/country/rwanda/overview>. 2. World Health O. Primary health care systems (primasys): case study from Rwanda: abridged version. Primary health care systems (primasys): case study from Rwanda: abridged version2017. |
| Papua New Guinea | 1. Institute PILI. Papua New Guinea Consolidated Legislation: Public Hospitals (Charges) Regulation 1978. Online; 2022. Available from: <http://www.paclii.org/pg/legis/consol_act/phr1978333/> [Last Accessed; 9th Dec]. 2. Independent State of Papua New Guinea. Public Hospitals Act 1994. 2022. Available from: <http://www.paclii.org/pg/legis/consol_act/pha1994188.pdf#:~:text=AN%20ACT%20entitled%20Public%20Hospitals%20Act%201994%2C%20Being,their%20powers%20and%20functions%2C%20and%20for%20related%20purposes>. [Last Accessed; 10th Dec]. 3. Government of Papua New Guinea. The Alotau Accord – 2012 Summary of O’Neill Government Priorities. Online; 2022. Available from: <https://www.dfat.gov.au/about-us/publications/Pages/papua-new-guinea-alotau-accord-summary-oneill-gov-priorities> [Last Accessed; 10th Dec]. 4. Government of Papua New Guinea. Constitution of the Independent State of Papua New Guinea, National Goals and Directive Principles [Online]. 5. Government of Papua New Guinea. National Health Plan. Online; 2021. 6. Government of Papua New Guinea. National Department of Health. National Health Services Standards: Annex 1;Application of Quality Standards for Health Services in Papua New Guinea. 2011. 7. Department of Health. Coronavirus disease 2019 (COVID-19):  Papua New Guinea Situation Report 15 Online; 2020. 8. International Health Facility Guidelines Part B - Appendix A – Role Delineation Level Guide[Online]. 2014. | 1. Hou X, Khan MM, Pulford J, et al. Readiness of health facilities to provide emergency obstetric care in Papua New Guinea: evidence from a cross-sectional survey. BMJ Open 2022;12(2):e050150, doi:10.1136/bmjopen-2021-050150 2. World Bank. Population, total- Papua New Guinea. Online; 2022. Available from: <https://data.worldbank.org/indicator/SP.POP.TOTL?locations=PG> [Last Accessed; 10th Dec]. 3. World Bank. Country and Lending Groups \| Data[Online]. 2016. Available from: <http://data.worldbank.org/about/country-and-lending-groups> [Last Accessed; 7th May]. 4. Grundy J, Dakulala P, Wai K, et al. Papua New Guinea Health System Review [Accessed Online]. World Health Organization, Regional Office for South-East Asia: New Delhi; 2019. 5. Asian Development. Revitalizing Rural Health Services in Papua New Guinea \| Asian Development Blog. 2022. Available from: <https://blogs.adb.org/blog/revitalizing-rural-health-services-png> [Last Accessed; 10th Dec]. 6. World Health Organisation. Country Cooperation Strategy: Papua New Guinea. 2018. |
| Myanmar | 1. The Republic of the Union of Myanmar. The Social Security Law. 2012. 2. Ministry of Labour of the Republic of the Union of Myanmar. Report on Myanmar labour force survey 2015. 2015. 3. Ministry of Health of the Republic of the Union of Myanmar. National health insurance implementation committee. 2022. 4. Ministry of Health of the Republic of the Union of Myanmar. Myanmar Health Care System. In: Health in Myanmar 2014. pp. 6–8, 2014, Ministry of Health, Nay Pyi Taw, Myanmar. 5. Ministry of Health of the Republic of the Union of Myanmar. Health in Myanmar 2014 [Internet]. 2014. Available from: [www.moh.gov.mm](http://www.moh.gov.mm/) 6. Ministry of Health of the Republic of the Union of Myanmar. [Internet]. [cited 2022 Oct 25]. Available from: https://[www.mohs.gov.mm/Main/content/page/organization-](http://www.mohs.gov.mm/Main/content/page/organization-) chart 7. Ministry of Health of the Republic of the Union of Myanmar. Myanmar demographic and health survey 2015- 2016 [Internet]. 2017 [cited 2022 Jul 26]. Available from: https://dhsprogram.com/pubs/pdf/FR324/FR324.pdf 8. Ministry of Health of the Republic of the Union of Myanmar. National health plan (2017-2021). 2017. 9. Ministry of Labour of the Republic of the Union of Myanmar. The 2014 Myanmar population and housingcensus [Internet]. 2017. Available from: [www.dop.gov.mm](http://www.dop.gov.mm/) 10. Ministry of Health of the Republic of the Union of Myanmar. Myanmar health statistics [Internet]. 2020 [cited 2022 Jul 26]. Available from: <https://mohs.gov.mm/Main/content/publication/myanmar-health-statistics-2020> 11. Ministry of Immigration and Population of the Republic of the Union of Myanmar. Myanmar 2019 Inter-censal survey: Union report [Internet]. 2020 [cited 2022 Jul 26]. Available from: <https://dop.gov.mm/sites/dop.gov.mm/files/publication_docs/ics_report_eng_7012021.pdf> 12. Myanmar National Portal. Ethnic groups in Myanmar [Internet]. 2022 [cited 2022 Jul 20]. Available from: https://myanmar.gov.mm/news-media/news/latest-news/-   /asset_publisher/idasset354/content/moken-or-salon-people-living-in-the-far-south- of-myanmar | 1. World Health Organisation. The world health report 2006: working together for health. World Health Organization; 2006. 209 p. 2. Mon Saw Y, Mon Than T, Thaung Y, Aung S, Wen-Shuan Shiao L, Mon Win E, et al. Myanmar’s human resources for health: current situation and its challenges. Heliyon [Internet]. 2019;5:1390. Available from: https://doi.org/10.1016/j.heliyon.2019.e01390 3. Aung MN, Shiu C, Chen WT. Amid political and civil unrest in Myanmar, health services are inaccessible. Vol. 397, The Lancet. Elsevier B.V.; 2021. p. 1446. 4. BBC news. Myanmar coup: The doctors and nurses defying the military [Internet]. 2021 [cited 2022 Jul 17]. Available from: https://[www.bbc.co.uk/news/world-asia-](http://www.bbc.co.uk/news/world-asia-)59649006 5. Sin H, Jewelwayne T, Cain S. Myanmar health financing system assessment [Internet]. 2018 [cited 2022 Jul 27]. Available from: https://documents1.worldbank.org/curated/en/506281543467798250/pdf/132560- 28-11-2018-13-35-16-MyanmarHFSAFINAL.pdf 6. The World Bank. Domestic general government health expenditure (% of general government expenditure) - Myanmar \| Data [Internet]. 2022 [cited 2022 Jul 20]. Available from: https://data.worldbank.org/indicator/SH.XPD.GHED.GE.ZS?locations=MM 7. The World Bank. Out-of-pocket expenditure (percent of current health expenditure): Myanmar [Internet]. 2022 [cited 2022 Jul 20]. Available from: https://data.worldbank.org/indicator/SH.XPD.OOPC.CH.ZS?locations=MM 8. World Health Organisation. Global expenditure on health: Public spending on the rise? [Internet]. 2022 [cited 2022 Jul 26]. Available from: <https://apps.who.int/iris/rest/bitstreams/1400583/retrieve> 9. International Labour Organization., ILO Liaison Officer for Myanmar. Social protection within the framework of labour legislation reform in Myanmar : background research summary [Internet]. ILO; 2015 [cited 2022 Jul 27]. 25 p. Available from: https://[www.ilo.org/wcmsp5/groups/public/---asia/---ro-bangkok/---ilo-](http://www.ilo.org/wcmsp5/groups/public/---asia/---ro-bangkok/---ilo-) yangon/documents/publication/wcms_375564.pdf 10. Asia Development Bank PEO. Country synthesis of post-evaluation findings, Myanmar. 1996. 11. International Labour Organisation. Social health protection: Social security board reforms: Enhance and extend health protection in Myanmar [Internet]. 2020 [cited 2022 Jun 23]. Available from: https://[www.ilo.org/yangon/press/WCMS_736183/lang--en/index.htm](http://www.ilo.org/yangon/press/WCMS_736183/lang--en/index.htm) 12. Myint CY, Pavlova M, Groot W. Catastrophic health care expenditure in Myanmar: Policy implications in leading progress towards universal health coverage. Int J Equity Health. 2019 Jul 30;18(1). 13. Chit Su Tinn. Towards universal health coverage in Myanmar: A policy alignment study. 2022. 14. International Labour Organisation., ILO Liaison Officer for Myanmar. Evaluation of the operations of the Social Security Board : ILO-MDRI technical report. ILO; 2015. 56 p. 15. FIND. Myanmar healthcare profile. 2017. 16. Huang F, Chen WT, Shiu CS, Lin SH, Tun MS, Nwe TW, et al. Adaptation and validation of a culturally adapted HIV stigma scale in Myanmar. BMC Public Health [Internet]. 2021 Dec 1 [cited 2022 Jul 26];21(1). Available from: https://bmcpublichealth.biomedcentral.com/articles/10.1186/s12889-021-11685-w 17. Lemons-Lyn A, Reidy W, Myint WW, Chan KN, Abrams E, Aung ZZ, et al. Optimising HIV services for key populations in public-sector clinics in Myanmar. J Int Assoc Provid AIDS Care [Internet]. 2021 Nov 1 [cited 2022 Jul 26];20. Available from: https://[www.ncbi.nlm.nih.gov/pmc/articles/PMC8640295/pdf/10.1177_2325958221](http://www.ncbi.nlm.nih.gov/pmc/articles/PMC8640295/pdf/10.1177_2325958221) 1055933.pdf 18. Latt NN, Cho SM, Mie N, Htun M, Mon Saw Y, Noe M, et al. Healthcare in Myanmar [Internet]. Vol. 78, Nagoya J. Med. Sci. 2016. Available from: [http://www.moh.gov.mm](http://www.moh.gov.mm/) 19. Lwin KZ, Punpuing S. Determinants of institutional maternity services utilisation in Myanmar. PLoS One [Internet]. 2022 Apr 1 [cited 2022 Jul 26];17(4 April). Available from: https://[www.ncbi.nlm.nih.gov/pmc/articles/PMC9037929/pdf/pone.0266185.pdf](http://www.ncbi.nlm.nih.gov/pmc/articles/PMC9037929/pdf/pone.0266185.pdf) 20. World Bank. Maternal mortality ratio (modeled estimate, per 100,000 live births) - Myanmar \| Data [Internet]. 2022 [cited 2022 Jul 20]. Available from: https://data.worldbank.org/indicator/SH.STA.MMRT?locations=MM 21. World Bank. Mortality rate, under-5 (per 1,000 live births) - Myanmar \| Data [Internet]. 2022 [cited 2022 Jul 20]. Available from: https://data.worldbank.org/indicator/SH.DYN.MORT?locations=MM 22. World Bank Country and Lending Groups – World Bank Data Help Desk [Internet]. [cited 2022 Oct 20]. Available from: https://datahelpdesk.worldbank.org/knowledgebase/articles/906519-world-bank- country-and-lending-groups 23. Sein TT, Viroj Tangcharoensathien, Walaiporn Patcharanarumol, Asia Pacific Observatory on Health Systems and Policies. The Republic of the Union of Myanmar health system review [Internet]. 2014 [cited 2022 Jul 27]. 124 p. Available from: https://apps.who.int/iris/bitstream/handle/10665/208211/9789290616665_eng.pdf? sequence=1&isAllowed=y 24. World Health Organisation. Myanmar: Country Data [Internet]. 2020 [cited 2022 Mar 5]. Available from: https://[www.who.int/myanmar/](http://www.who.int/myanmar/) |
| Burundi | 1. Ministere de la Sante Publique et de la Lutte Contre le Sida. Normes Sanitaires pour la mise en oevre de la Strategie Sectorielle 2021-2027: Tome 1. Burundi. 2022. 2. Ministere de la Sante Publique et de la Lutte Contre le Sida. Resume analytique du profil sanitaire du Burundi. Burundi. 2021. 3. Ministere de la Sante Publique et de la Lutte Contre le Sida. Strategie Sectorielle de la Sante 2021-2027. Burundi. 2021. 4. Ministere de la Sante Publique et de la Lutte Contre le Sida. Guide operationnel de gestion du district sanitaire au Burundi. Burundi. 2020. 5. Ministere de la Sante Publique et de la Lutte Contre le Sida. Normes Sanitaires pour la mise en oevre de la Strategie Sectorielle 2021-2027: Tome 2. Burundi. 2022. | 1. The World Bank. Burundi: Overview 2022 [Available from: <https://data.worldbank.org/country/burundi>. 2. The World Bank. World Bank Country and Lending Groups 2023 [cited 2022 01/12/2022]. Available from: <https://datahelpdesk.worldbank.org/knowledgebase/articles/906519-world-bank-country-and-lending-groups>. 3. The World Bank. Hospital beds (per 1,000 people)- Burundi 2014 [Available from: <https://data.worldbank.org/indicator/SH.MED.BEDS.ZS?locations=BI&view=chart>. 4. Statoids. Burundi Country Profile 2022 [Available from: <https://www.statoids.org/en/country-profile/burundi>. 5. Fabrice Iranzi. Key facts you should know about the Burundi Health System. Region Week. 2022 8/12/2022. 6. Georges Nsengiyumva, Laurent Musango. The simultaneous introduction of the district health system and performance-based funding: the Burundi experience. Field Actions Science Reports. 2013(8) |
| Nepal | 1. Government of Nepal, *Nepal Health Facility Survey 2021: Final Report*, Ministry of Health and Population, Editor. 2022: Nepal. 2. Government of Nepal, *National Health sector strategy – implementation plan 2016-2021*, Ministry of Health and Population, Editor. 2016: Nepal. 3. Government of Nepal, *Annual Report 2020/2021*, Department of Health Services, Editor. 2021: Nepal. 4. Government of Nepal, *National Health sector strategy – implementation plan 2016-2021*, Ministry of Health and Population, Editor. 2016: Nepal. 5. Government of Nepal, *Annual Report 2020/2021*, Department of Health Services, Editor. 2021: Nepal. 6. Government of Nepal, *Nepal National Health Accounts 2012/2013- 2015/2016*, Ministry of Health and Population, Editor. 2018. 7. Government of Nepal, *Nepal Health Sector Strategy 2015-2020*, Ministry of Health and Population, Editor. 2015. 8. Government of Nepal, *Review of National Health Policy 1991*, Ministry of Health and Population, Editor. 2012 9. Government of Nepal, *The Public Health Service Act, 2075 (2018)*, Law Commission, Editor. 2018. 10. Government of Nepal, *National Health Policy 2019: English Version*, Ministry of Health and Population, Editor. 2021. 11. Government of Nepal, *Publish Health Service Regulation 2020: Unofficial Translation*, Ministry of Health and Population, Editor. 2020. | 1. World Bank. *Nepal, Population*. 2021 [cited 2022 11/11/2022]; Available from: <https://data.worldbank.org/indicator/SP.POP.TOTL?locations=NP>. 2. World Bank. *Nepal, Lower middle income*. 2022. 3. World Bank. *Nepal, Hospital Beds*. 2012. 4. UNRCO. *Nepal: Administrative Unit Map*. 2022 [cited 2022 16/12/2022]; Available from: <https://un.info.np/Net/NeoDocs/View/8225>. 5. Shiba K Rai, et al., *The Health System in Nepal — An Introduction.* Environmental Health and Preventive Medicine, 2001(6) 6. WHO, *WHO Nepal: Country Cooperation Strategy at a Glance*. 2018. 7. Jenna Wright, *Essential Health Services: Nepal*. 2015, USAID, 8. Government of Nepal, *The Interim Constitution of Nepal 2063 (2007)*. 2007. 9. Ministry of Health, *Nepal Health Sector Support Programme III (NHSSP – III): Report on Stocktaking the Health Policies of Nepal*. 2018: Nepal. 10. Government of Nepal, *Social Health Security Program (Health Insurance)*, S.H.S.D. Committee, Editor. 2017. 11. Himalayan News Service, *Four categories of hospitals in federal setup*, in *The Himalayan Times*. 2017 12. Adhikari, B., S.R. Mishra, and R. Schwarz, *Transforming Nepal's primary health care delivery system in global health era: addressing historical and current implementation challenges.* Global Health, 2022. 18(1): p. 8. 13. Thapa, N.R., S. Adhikari, and P.K. Budhathoki, *Influence of internal migration on the use of reproductive and maternal health services in Nepal: An analysis of the Nepal Demographic and Health Survey 2016.* PLoS One, 2019. 14(5): p. e0216587. |
| Sri-Lanka | 1. *Statistical Data Sheet 2021*. 2021, Department of Census and Statistics: Sri Lanka. 2. *No of GN by DS*. 2011, Department of Census and Statistics: Sri Lanka 3. *Piloting a Primary Health Care Reform in Sri Lanka: A guide to establishing a ‘cluster of facilities’ for providing shared care*. 2019, Ministry of Health Nutrition & Indigenous Medicine and The Asian Development Bank: Sri Lanka. 4. *Manual of management of Divisional hospitals*. 1994, Ministry of Health and Women's Affairs: Sri Lanka. 5. *Reorganising Primary Health Care in Sri Lanka: Preserving our progress, preparing our future*. 2017, Ministry of Health Nutrition and Indigenous Medicine: Sri Lanka. 6. *Sri Lanka Essential Health Services Package*, M.o.H.N.a.I. Medicine, Editor. 2019: Sri Lanka | 1. Smith, O. 2018. “Sri Lanka: Achieving Pro-Poor Universal Health Coverage without Health Financing Reforms”. Universal Health Coverage Study Series No. 38, World Bank Group, Washington, DC. 2. Serajuddin, U. and N. Hamadeh, *New World Bank country classifications by income level: 2020-2021*, in *World Bank Blogs*. 2020. 3. Rajapaksa, L., et al., *Sri Lanka health system review*. 2021, World Health Organization Regional Office for South-East Asia: New Delhi. 4. Rannan-Eliya, R. and L. Sikurajapathy, *Sri Lanka: “Good Practice” in Expanding Health Care Coverage*. 2008, Institute for Health Policy: Colombo, Sri Lanka. 5. *Improving retention of health workers in rural and remote areas: Case studies from WHO South-East Asia Region*. 2020, WHO South-East Asia Region: Delhi, India. 6. Kumar, R., *Public–private partnerships for universal health coverage? The future of “free health” in Sri Lanka.* Globalization and Health, 2019. 15(1): p. 1-10. 7. Sri Balakrishnan, S. and M. Caffrey, *Policy brief for Sri Lanka*. 2022, UNICEF. 8. Susie Perera, S., et al., *Accelerating reforms of primary health care towards universal health coverage in Sri Lanka.* Perspective, 2019. 8(1) 9. WHO. *Global Health Workforce statistics database*. 2022 [cited 2022 29/07/2022]; Available from: <https://www.who.int/data/gho/data/themes/topics/health-workforce>. 10. De Silva, D., *How many doctors should we train for Sri Lanka? System dynamics modelling for training needs.* Ceylon Medical Journal, 2017. 62: p. 233-37. 11. De Silva, V., et al., *The assistant medical officer in Sri Lanka: mid-level health worker in decline.* J Interprof Care, 2013. 27(5): p. 432-3. |

**Supplementary Material 5: Case study MS Word template**

| Country |  |
| --- | --- |
| Population size |  |
| Income group |  |
| Hospital beds/population |  |
| Administrative structure |  |
| Brief background |  |
| Healthcare administration |  |
| Financing of hospital care |  |
| Levels and types of hospitals |  |
| Diagram of hospital care hierarchy |  |
| Key timeline of policy changes |  |
| Identified issues affecting hospital care |  |
| Does primary care concept include hospital care? |  |
| Contextual first referral level hospital |  |
| Defined minimum service package and staffing norms |  |

**Supplementary Material 6: Summary of hospital related World Health Assembly Resolutions and Decisions, 2007-2021**

| **Date** | **Resolutions related to hospitals** | **Resolutions related to FRH** |
| --- | --- | --- |
| 2007(WHO, 2007) | Health systems: emergency-care systems; urges member states to assess comprehensively the prehospital and emergency-care context including, where necessary, identifying unmet needs; and to consider establishing formal and integrated emergency-care systems and to draw on informal systems and community resources in order to establish integrated-care capacity in areas where formal, prehospital, emergency medical-care systems are impractical; and  Requests director general to devise standardized tools and techniques for assessing need for prehospital and facility-based capacity in trauma and emergency care; | None |
| 2008(WHO, 2008) | None | None |
| 2009(WHO, 2009) | None | None |
| 2010(WHO, 2010) | Infant and young child nutrition  Urges member states to strengthen and expedite the sustainable implementation of the global strategy for infant and young child feeding including the implementation of the Baby-friendly Hospital Initiative  AND  Accelerated progress towards achievement of Millennium Development Goal 4 to reduce child mortality: prevention and treatment of pneumonia:  Urges member states to implement the recommendations in the joint WHO/UNICEF global action plan for the prevention and control of pneumonia, including integrated case management at community, health-centre and hospital levels (etc). | None |
| 2011(WHO, 2011) | To strengthen national health emergency and disaster management capacities and the resilience of health systems, urges member states to develop programmes on safe and prepared hospitals that ensure that new hospitals and health facilities are located and built safely so as to withstand local hazards; that the safety of existing facilities is assessed and remedial action is taken; and that all health facilities are prepared to respond to internal and external emergencies;  AND  Working towards the reduction of perinatal and neonatal mortality: urges member states  to further promote political commitment for effective implementation of the existing national, regional and/or global plans with the application of evidence-based strategies and interventions, including the Baby-Friendly Hospital Initiative, to improve perinatal and neonatal health and increase equitable access to quality maternal, newborn and child health services | None |
| 2012(WHO, 2012) | None | None |
| 2013(WHO, 2013) | None | None |
| 2014(WHO, 2014) | Antimicrobial resistance:  Urges member states to develop antimicrobial resistance surveillance systems in three separate sectors: (i) inpatients in hospitals, (ii) outpatients in all other health care settings and the community and (iii) animals and non-human usage of antimicrobials; | To improve the quality of maternal and newborn care:  Staffing levels for each facility providing maternal and newborn care need to be planned in such a way that services can be provided on a continuous basis, 24 hours a day, seven days a week. Teams in first- and second-level referral hospitals should be multidisciplinary include specialized obstetric, paediatric and anaesthetic staff to manage maternal and neonatal complications. |
| 2015(WHO, 2015) | None | To strengthen emergency and essential surgical care and anaesthesia as a component of universal health coverage, urges member states to identify and prioritize a core set of emergency and essential surgery and anaesthesia services at the primary health care and first-referral hospital level, and to develop methods and financing systems for making quality, safe, effective and affordable emergency and essential surgical care and anaesthesia services accessible to all who need them, including promoting timely referral and more effective use of the health care workforce through task-sharing, as appropriate, as part of an integrated surgical care network in order to achieve universal health coverage; (2) to integrate emergency and essential surgical care and anaesthesia in primary health care facilities and first-referral hospitals, and to promote emergency and essential surgery and anaesthesia capacity as components integral to achieving universal health coverage; |
| 2016(WHO, 2016) | Addressing the challenges of the United Nations Decade of Action for Road Safety (2011–2020): outcome of the second Global High-level Conference on Road Safety – Time for Results:  Requests the director general to provide technical support for the strengthening of pre-hospital care, including emergency health services and the immediate post-crash response, hospital and ambulatory guidelines for trauma care, and rehabilitation services, capacity building and improvement of timely access to integral health care; | None |
| 2017(WHO, 2017) | None | None |
| 2018(WHO, 2018) | Infant and young child feeding:  Urges member states to reinvigorate the Baby-friendly Hospital Initiative | None |
| 2019(WHO, 2019) | Emergency care systems for universal health coverage: ensuring timely care for the acutely ill and injured:  Urges member states to work towards, or promote the inclusion of routine prehospital and hospital emergency unit care within health strategies, and within other relevant planning documents; and to develop a governance mechanism for the coordination of routine prehospital and hospital-based emergency care services; and to support efforts to ensure, based on local risks, that prehospital and hospital emergency units have plans in place to protect providers, patients and infrastructure from violence and to protect providers and patients from discrimination; and that they have in place clear protocols for the prevention and management of hazardous exposures | None |
| 2020(WHO, 2020) | None | None |
| 2021(WHO, 2021) | None | None |

**References for supplementary material**

Cochrane Effective Practice and Organisation of Care. (2020). *LMIC Filters*. Retrieved 04/08/2022 from <https://epoc.cochrane.org/lmic-filters>

The Lancet. (2022a). *Commissions from the Lancet journals*. <https://www.thelancet.com/commissions>

The Lancet. (2022b). *Series from the Lancet journals*. <https://www.thelancet.com/series>

WHO. (2007). *SIXTIETH WORLD HEALTH ASSEMBLY: RESOLUTIONS AND DECISIONS ANNEXES*. <https://apps.who.int/gb/ebwha/pdf_files/WHASSA_WHA60-Rec1/E/WHASS1_WHA60REC1-en.pdf>

WHO. (2008). *SIXTY-FIRST WORLD HEALTH ASSEMBLY: RESOLUTIONS AND DECISIONS ANNEXES*. <https://apps.who.int/gb/ebwha/pdf_files/WHA61-REC1/A61_REC1-en.pdf>

WHO. (2009). *SIXTY-SECOND WORLD HEALTH ASSEMBLY: RESOLUTIONS AND DECISIONS ANNEXES*. <https://apps.who.int/gb/ebwha/pdf_files/WHA62-REC1/WHA62_REC1-en.pdf>

WHO. (2010). *SIXTY-THIRD WORLD HEALTH ASSEMBLY: RESOLUTIONS AND DECISIONS ANNEXES* <https://apps.who.int/gb/ebwha/pdf_files/WHA63-REC1/WHA63_REC1-en.pdf>

WHO. (2011). *SIXTY-FOURTH WORLD HEALTH ASSEMBLY: RESOLUTIONS AND DECISIONS ANNEXES*. <https://apps.who.int/gb/ebwha/pdf_files/WHA64-REC1/A64_REC1-en.pdf>

WHO. (2012). *SIXTY-FIFTH WORLD HEALTH ASSEMBLY: RESOLUTIONS AND DECISIONS ANNEXES*. <https://apps.who.int/gb/ebwha/pdf_files/WHA65-REC1/A65_REC1-en.pdf>

WHO. (2013). *SIXTY-SIXTH WORLD HEALTH ASSEMBLY: RESOLUTIONS AND DECISIONS ANNEXES*. <https://apps.who.int/gb/ebwha/pdf_files/WHA66-REC1/A66_REC1-en.pdf>

WHO. (2014). *SIXTY-SEVENTH WORLD HEALTH ASSEMBLY: RESOLUTIONS AND DECISIONS ANNEXES*. <https://apps.who.int/gb/ebwha/pdf_files/WHA67-REC1/A67_2014_REC1-en.pdf#page=1>

WHO. (2015). *SIXTY-EIGHTH WORLD HEALTH ASSEMBLY: RESOLUTIONS AND DECISIONS ANNEXES*. <https://apps.who.int/gb/ebwha/pdf_files/WHA68-REC1/A68_R1_REC1-en.pdf#page=1>

WHO. (2016). *SIXTY-NINTH WORLD HEALTH ASSEMBLY: RESOLUTIONS AND DECISIONS ANNEXES*. <https://apps.who.int/gb/ebwha/pdf_files/WHA69-REC1/A69_2016_REC1-en.pdf#page=1>

WHO. (2017). *SEVENTIETH WORLD HEALTH ASSEMBLY: RESOLUTIONS AND DECISIONS ANNEXES*. <https://apps.who.int/gb/ebwha/pdf_files/WHA70-REC1/A70_2017_REC1-en.pdf#page=1>

WHO. (2018). *SEVENTY-FIRST WORLD HEALTH ASSEMBLY: RESOLUTIONS AND DECISIONS ANNEXES*. <https://apps.who.int/gb/ebwha/pdf_files/WHA71-REC1/A71_2018_REC1-en.pdf#page=1>

WHO. (2019). *SEVENTY-SECOND WORLD HEALTH ASSEMBLY: RESOLUTIONS AND DECISIONS ANNEXES*. <https://apps.who.int/gb/ebwha/pdf_files/WHA72-REC1/A72_2019_REC1-en.pdf#page=1>

WHO. (2020). *SEVENTY-THIRD WORLD HEALTH ASSEMBLY: RESOLUTIONS AND DECISIONS ANNEXES*. <https://apps.who.int/gb/ebwha/pdf_files/WHA73-REC1/A73_REC1-en.pdf#page=1>

WHO. (2021). *SEVENTY-FOURTH WORLD HEALTH ASSEMBLY: RESOLUTIONS AND DECISIONS ANNEXES*. <https://apps.who.int/gb/ebwha/pdf_files/WHA74-REC1/A74_REC1-en.pdf#page=1>
